# Cellular mechanosignaling in pulmonary arterial hypertension

**DOI:** 10.1007/s12551-021-00828-3

**Published:** 2021-09-02

**Authors:** Ariel Wang, Daniela Valdez-Jasso

**Affiliations:** Bioengineering Department, University of California San Diego, La Jolla, CA USA

**Keywords:** Mechanosignaling, Pulmonary arterial endothelial cells, Pulmonary arterial smooth muscle cells, Pulmonary arterial adventitial fibroblasts, Stiffness, Stretch, Computational model

## Abstract

Pulmonary arterial hypertension (PAH) is a vasculopathy characterized by sustained elevated pulmonary arterial pressures in which the pulmonary vasculature undergoes significant structural and functional remodeling. To better understand disease mechanisms, in this review article we highlight recent insights into the regulation of pulmonary arterial cells by mechanical cues associated with PAH. Specifically, the mechanobiology of pulmonary arterial endothelial cells (PAECs), smooth muscle cells (PASMCs) and adventitial fibroblasts (PAAFs) has been investigated in vivo, in vitro, and in silico. Increased pulmonary arterial pressure increases vessel wall stress and strain and endothelial fluid shear stress. These mechanical cues promote vasoconstriction and fibrosis that contribute further to hypertension and alter the mechanical milieu and regulation of pulmonary arterial cells.

## Introduction

### Pulmonary arterial hypertension

Pulmonary arterial hypertension (PAH) is a vasculopathy that is manifested by sustained elevation of pulmonary arterial pressures and irreversible vascular remodeling. In PAH, the pulmonary vasculature undergoes significant structural and functional remodeling, including thrombus formation, endothelial dysfunction, cell proliferation, migration, and hypertrophy, and accumulation of extracellular matrix (ECM) proteins, leading to the formation of complex lesions known as plexiform lesions (Ogata and Iijima 1993). As a consequence, there is thickening of the vascular wall, persistent vasoconstriction, arterial stiffening, and vascular rarefaction that further exacerbate pressure overload and adversely impairs pulmonary artery (PA) perfusion and hemodynamics.

Therapeutics targeting different cell types involved in this cascade of events have been developed to ameliorate the symptoms or progression of PAH. Vasodilators targeting smooth muscle cells by stimulating nitric oxide (NO) release have shown to reduce pulmonary arterial pressure, but do not reverse adverse vascular remodeling (Sun and Chan 2018). Other therapeutic targets include G protein-coupled receptors (GPCRs), ion channels, metabolic pathways, transcription factors, and growth factor receptors (Hemnes and Humbert 2017; Sommer et al. 2021). NO release is stimulated by phosphodiesterase type 5 (PDE5) inhibitors tadalafil and sildenafil, and endothelin receptor antagonists ambrisentan and macitentan also lead to smooth muscle cell relaxation. Bone morphogenic protein receptor type 2 (BMPR2) antagonists like chloroquine and Smad2/3 antagonists like sotatercept are used to target the BMP and transforming growth factor beta (TGF-β1) pathways, respectively (Sommer et al. 2021). Since the structural and cellular remodeling of the pulmonary arteries is still largely irreversible, the prognosis of PAH is poor, and the underlying causes remain untreatable. Existing drugs do not reduce the progression of vascular remodeling, and patients deteriorate over time. Fewer than 60% of patients survive more than five years after diagnosis (Dannewitz Prosseda et al. 2020) and lung transplantation remains the only cure (Sommer et al. 2021). Since PAH therapeutics have had limited efficacy in reversing the pathological mechanisms that drive vascular remodeling, there is a need for more research into the pathology and crosstalk between biochemical and mechanosensitive signaling pathways in pulmonary arterial cells (Sitbon et al. 2019).

## Cellular mechanical regulation

**Fig. 1:**
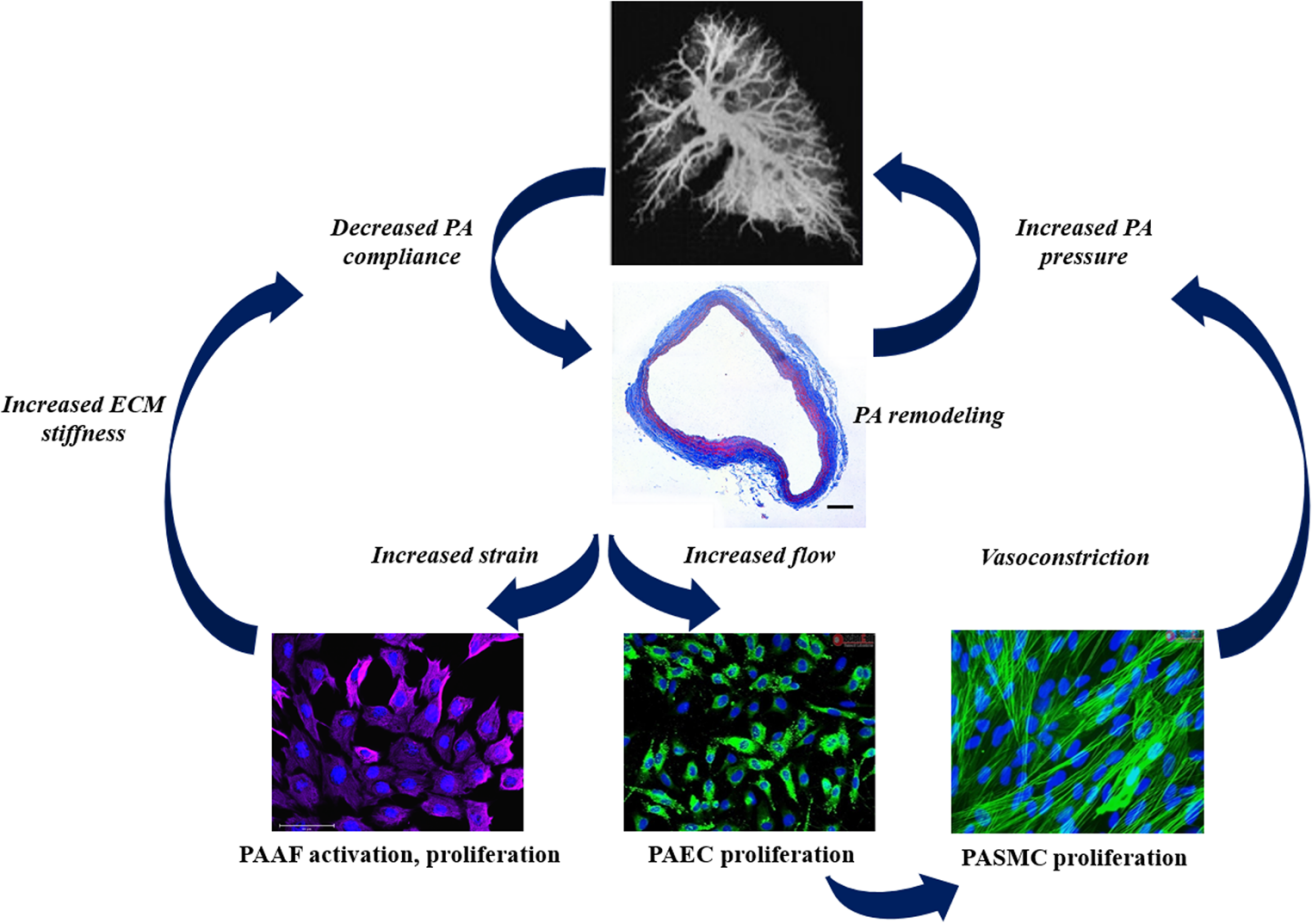
Vascular remodeling in pulmonary arterial hypertension is manifested by increased pulmonary arterial pressures, decreased arterial compliance, and pruning of the vasculature. At the cellular level, pulmonary arterial adventitial fibroblasts (PAAFs) respond to increased strain, endothelial cells (PAECs) respond to increased flow, and smooth muscle cells (PASMCs) are activated to contract causing vasoconstriction. These cellular responses feedback into the system by increasing extracellular matrix (ECM) stiffness, releasing paracrine factors, and increasing PA pressure, respectively. Images used with permission from J Signal Transduct. 2012: 951497, and sciencellonline.com

In response to the rise in mean pulmonary arterial pressure, the pulmonary artery wall thickens due to increased proliferation and hypertrophy of PA smooth muscle cells (PASMCs) (Shimoda and Laurie 2013; Tuder et al. 2013). This is accompanied by increased proliferation of PA endothelial cells (PAECs) and adventitial fibroblasts (PAAFs) as well as inhibited apoptosis of PAECs and PASMCs, and an endothelial-to-mesenchymal-transition (EndoMT), that transforms PAECs to myofibroblasts, leading to overproduction of ECM proteins and fibrosis (Good et al. 2015). The mechanical forces that trigger vascular cell remodeling in PAH result from the effect of increased blood pressure on the wall and increased blood flow on the endothelium (Kumar et al. 2010; Bertero et al. 2018). Increases in arterial pressure lead to vessel thickening, while increased PA flow due to PAH also increases PAEC fluid shear stress which promotes EndoMT and further accumulation of PAAFs (Good et al. 2015). Increased vessel loading also promotes PAAF proliferation and activates increased ECM expression resulting in matrix stiffening that in turn activates myofibroblasts to produce more ECM, further decreasing vascular compliance (Bertero et al. 2018). This cascade of events further exacerbates adverse structural remodeling and vascular dysfunction (Fig. 1).

**Fig. 2:**
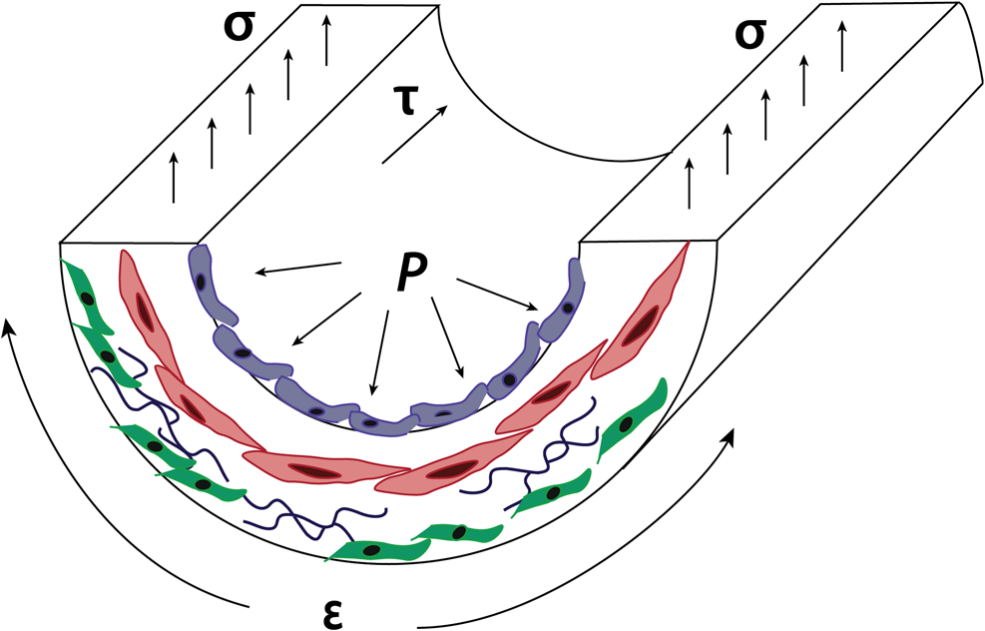
Increased pulmonary arterial pressure (*P*) increases vessel wall stress (*σ*), strain (*ε*), and blood flow, which increases shear fluid shear stress (*τ*) on the endothelium. The main cell types affected by these mechanical forces are PAECs — blue PASMCs — red, PAAFs — green

**Fig. 3:**
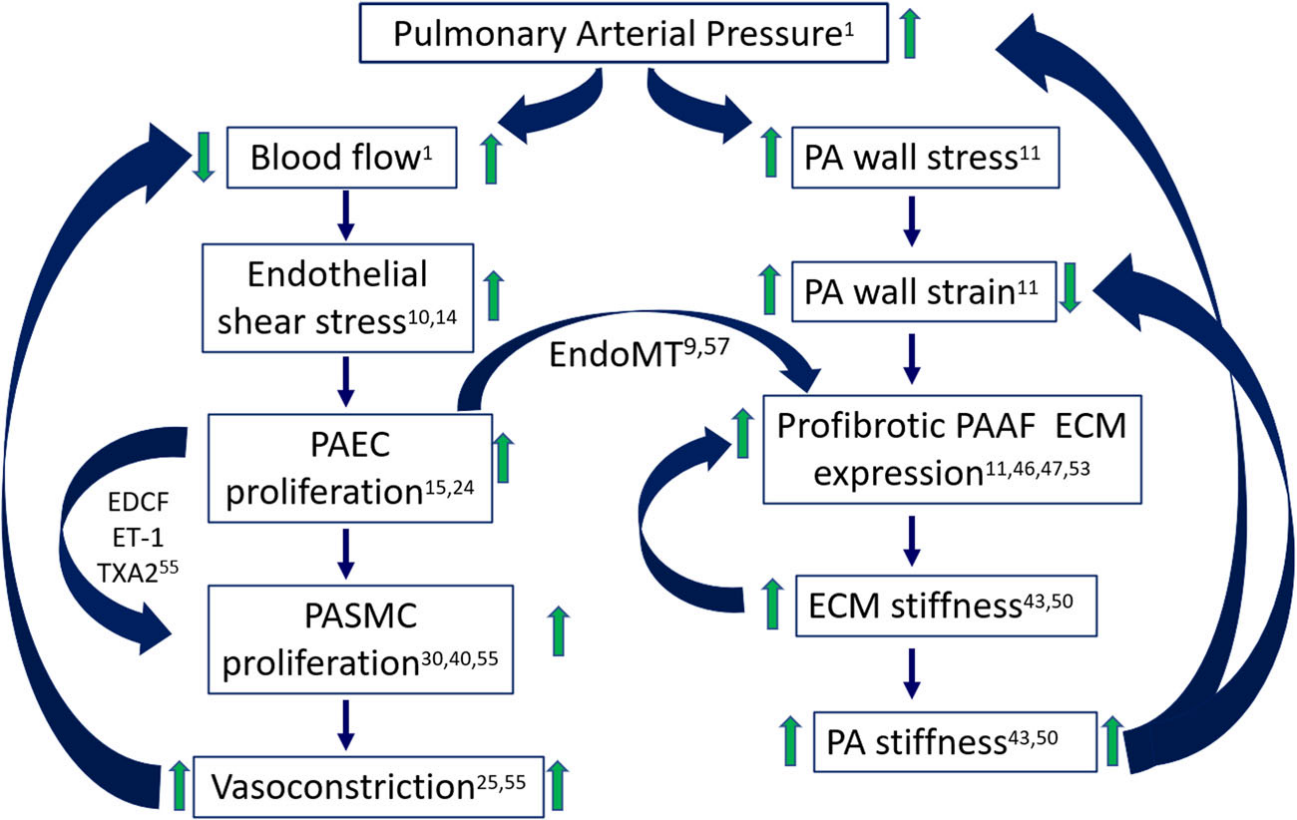
The effect of an increase of mean pulmonary arterial pressure (mPAP) on increasing blood flow results in increased shear stress experienced by pulmonary arterial endothelial cells (PAECs) which increases their proliferation. This and other cytokines mediate the PAEC transition into activated fibroblasts and release of paracrine factors that induce smooth muscle cell (PASMC) proliferation and vasoconstriction that in turn reduces blood flow. An increase in mPAP also increases PA wall stress and strain, which increases profibrotic adventitial fibroblast (PAAF) expression, increasing ECM stiffness and overall PA stiffness leading to decreased PA wall strain

Increased pulmonary arterial pressure causes increased vessel wall stress and strain and drives increased blood flow that increases fluid shear stress on the endothelium (Fig. 2). Changes in stiffness at the tissue scale of the pulmonary vasculature results in cellular mechanotransduction responses leading to activation of signaling pathways that feed back to induce more remodeling (Dieffenbach et al. 2018). For example, ECM gene expression by PAAFs can be activated by mechanical strain, leading to the overproduction of collagen. The resulting increase in ECM stiffness activates PAAFs to transition to myofibroblasts and can also stimulate PASMC and PAEC proliferation (Thenappan et al. 2018). Here we summarize the mechanobiological responses to wall stress and strain, fluid shear stress and ECM stiffening in these three cell types and the crosstalk between them in the context of vessel remodeling in response to mechanical overload in the pathogenesis of PAH (Fig. 3).

### Pulmonary arterial endothelial cells

PAECs form the permeable barrier between blood and vascular tissue, and respond to circulating cytokines such as endothelin-1 (ET-1), a vasoconstrictor, and platelet-derived growth factor (PDGF), which increases cell proliferation (Ranchoux et al. 2017; Welsh and Peacock 2013). PAEC signaling is also regulated by mechanical cues associated with altered fluid shear stress on the vascular endothelium due to increased blood pressure and flow, increases in matrix stiffness, and hypoxia. Dysregulation of pathways such as the vascular endothelial growth factor (VEGF), Notch, and bone morphogenetic protein (BMP) signaling pathways leads to disordered formation of new vessels (Ranchoux et al. 2017).

#### In-vivo experiments

Most experimental studies of vascular remodeling in PAH use one of three animal models: monocrotaline (MCT), chronic hypoxia (CH), and sugen-hypoxia (SuHx). MCT is a poisonous pyrolizidine alkaloid found in the leguminous plant *Crotalaria spectabilis*, that produces a massive inflammatory response in the lung, damaging pulmonary endothelial cells, leading to pathologically elevated pulmonary arterial pressures (Gomez-Arroyo 2017). Four weeks post-MCT injection in rodents is a well-established animal model of end-stage PAH. MCT is however toxic to the liver and other tissues (Gomez-Arroyo et al. 2012; Zhu et al. 2018), and commonly results in sudden cardiac death (Temple et al. 2014). Exposure to chronic hypoxia (10% oxygen) for few weeks induces pathologic elevation in pulmonary arterial pressure via PA medial hypertrophy and sustained pulmonary vasoconstriction (Liang et al. 2017). SuHx in rats, an animal model developed by Taraseviciene-Stewart et al. (Taraseviciene-Stewart et al. 2001), is the model of PAH that most closely recapitulates the pulmonary arterial lesions found in lung tissues from human PAH patients (Woodcock et al. 2021) and the progressive hemodynamic sequelae. With a single injection of 20 mg/kg of vascular endothelial growth receptor factor inhibitor sugen SU5416 and exposure to chronic hypoxia (10% oxygen) for three weeks, rats show significant pulmonary arterial medial hypertrophy, pulmonary arterial wall thickening and sustained pulmonary vasoconstriction that results in progressively elevated pressures (Nickel et al. 2015; Woodcock et al. 2021).

In both chronic hypoxia and MCT rats, hyper-proliferation of PAECs and increased levels of VEGF in vivo mediated vessel growth and disordered angiogenesis (Liang et al. 2017). Chronic hypoxia induces expression of PDGF, increasing PAEC proliferation and inhibiting apoptosis. This indicates that hypoxia-induced angiogenesis in PAECs stimulates elevated expression of PDGF and VEGF, growth factors that are well known to regulate vascular remodeling.

In the MCT rat model of PAH, downregulation of miR-371b-5p was shown to increase apoptosis of PAECs via phosphatase and tensin homolog (PTEN)/ phosphoinositide 3-kinase (PI3K)/Akt signaling and to suppress endothelial nitric oxide synthase (eNOS) synthesis of nitric oxide which is a vasodilator (Zhu et al. 2018). This miRNA could thus be an important regulator of proliferation of PAECs and vasoconstriction in PAH.

An important pathway that is dysregulated in PAH is BMP signaling. In SuHx rats and cultured lung samples from human patients, an elastase inhibitor Elafin was used to rescue BMP signaling, which is important in vasculogenesis, improving PAEC survival and normal angiogenesis and thereby reversing adverse remodeling (Nickel et al. 2015).

In a recent study by Woodcock et al., SuHx rats and lungs from PAH patients showed decreased miR-7 expression (Woodcock et al. 2021). miR-7 has been reported to regulate serine and arginine-rich splicing factor 1 (SRSF1), which promotes PAEC migration (Woodcock et al. 2021). Therefore, this may be a novel mechanism by which changes in ECM stiffness in PAH regulates pathologic endothelial dysfunction and cell migration.

#### In-vitro experiments

Increased fluid shear stress on PAECs in vitro has been shown to decrease Protein Kinase C delta (PKCδ) activity, which leads to increases in phosphorylation of eNOS and consequent generation of NO in primary ovine cell cultures (Kumar et al. 2010).

In primary human PAECs isolated from PAH patients, increased production of ET-1 was observed, which leads to PAEC dysfunction and excessive proliferation (Kang et al. 2016). Stimulation with peroxisome proliferator–activated receptor gamma (PPARγ) was able to reduce this ET-1 overexpression, demonstrating the potential for PPARγ agonists as therapeutics for PAH.

In vitro, increased extracellular matrix stiffness from 1 kilopascal (kPa), representing control arterioles, to 50 kPa, representing diseased pulmonary arterioles, led to increased glycolysis via the YAP/TAZ mechanotransducers in the Hippo signaling pathway (Bertero et al. 2016). A shift from oxidative phosphorylation to glycolysis is a driver of PAEC proliferation and migration early in PH, as glycolysis is needed to meet the metabolic demands of proliferating cells. When PAECs were grown on the aforementioned stiff 50-kilopascal (kPa) matrix representative of tissue stiffness in PAH compared with a soft 1-kPa matrix representative of control lungs by Woodcock et al., miR-7 was downregulated, which led to increased migration (Woodcock et al. 2021).

#### Summary

PAECs respond to the elevated ECM stiffness, fluid shear stress, and hypoxia associated with PAH via dysregulated PI3K and BMP signaling, which trigger hyper-proliferation and migration. This in turn mediates pathogenic angiogenesis and vascular remodeling.

### Pulmonary arterial smooth muscle cells

PASMCs are the primary cell type in the medial layer of the pulmonary artery. They contain contractile proteins which are regulated by calcium and control vascular tone (Lyle et al. 2017). When dysregulated, abnormal SMC contractility can cause persistent vasoconstriction, a disease marker of PAH. In PAH we also observe neointimal hyperplasia due to proliferation, hypertrophy, and migration of PASMCs to the intimal layer of the PA. Under PAH conditions, SMCs also produce more pro-inflammatory cytokines, deposit increased elastin and collagen, and grow thereby thickening the medial layer (Stenmark et al. 2018). SMCs respond to increases in matrix stiffness and hypoxia by remodeling the ECM and proliferating abnormally.

#### In-vivo experiments

Signaling pathways such as NFAT and Notch signaling become overactive in PAH conditions. In MCT rats, calcineurin/nuclear factor of activated T-cells (NFAT) signaling was reported to be activated and shown to increase PASMC proliferation and migration, and inhibit apoptosis (He et al. 2018). Notch signaling is another important pathway that upregulates proliferation and differentiation of PASMCs. The γ-secretase inhibitor DAPT blocks Notch3 and successfully reduced mean pulmonary arterial pressure in extrauterine growth restriction (EUGR) rats (Li et al. 2009; Wang et al. 2019).

Transient receptor potential vanilloid-3 (TRPV3) channel expression was also found to be increased in hypoxic rats, and is thought to mediate pulmonary vascular remodeling via the proliferation of PASMCs by activating PI3K/Akt signaling (Zhang et al. 2018).

There is evidence of regulation of PAH by noncoding RNAs. Upregulation of the newly found microRNA miR-205-5p was observed to reduce PASMC proliferation in a hypoxia-induced PAH mouse model, inhibiting molecule interacting with CasL 2 (MICAL2) expression by targeting the 3$^{\prime }$ untranslated region, which activates the ERK1/2 pathway (Tao et al. 2019). miRNAs were also found to be important in PASMC synthesis of collagen, particularly miR-29b, which physically binds Smad3 downstream of TGFβ1 as found by chromatin immunoprecipitation (Wang et al. 2018). TGF-β1 is an important cytokine because chronic activation in vivo leads to spontaneous PAH in mice (Calvier et al. 2019). TGF-β1 also downregulates miR-29b through Smad3 (Wang et al. 2018). PASMCs isolated from PAH patients by Lei et al. demonstrated an increase in a long noncoding RNA (lncRNA), which reduces the expression of miR-141 (Lei et al. 2020). miR-141 normally downregulates expression of RhoA, suppressing the RhoA/ROCK pathway, but when miR-141 is not highly expressed the ROCK pathway is upregulated, which increases constriction and remodeling of the vasculature.

#### In-vitro experiments

Rat PASMCs showed higher expression of collagen III protein and fibronectin mRNA when stimulated with connective tissue growth factor (CTGF), upstream of PI3K (Sun et al. 2017). This increase in ECM protein deposition promotes pulmonary vascular remodeling under PAH conditions.

Different pathways are upregulated by hypoxia in PASMCs. Huang et al. showed that the mitogen-activated protein kinases (MAPK) signal pathway is crucial in the proliferative response of PASMCs to hypoxia (Huang et al. 2017). Hypoxia induces pulmonary vasoconstriction, mediated by increased intracellular calcium in PASMCs (Yadav et al. 2018). Vasoconstriction causes reactive oxygen species (ROS)-dependent phospholipase C (PLCγ1) activation and contraction in mouse PASMCs.

BMP signaling is important to normal function of PASMCs but can be disrupted by disease. Wang et al. found in rat primary PASMCs that BMP signaling is suppressed in hypoxia-induced PH (Wang et al. 2019). Dysfunctional BMP signaling causes proliferation of PASMCs in PAH, and PDGF-BB can activate Rho kinase and enhance proliferation of rat SMCs (Wei et al. 2019).

NFAT signaling can also be dysregulated in PAH. PASMCs isolated from PAH patients and control subjects showed upregulated STIM2 in PAH-PASMCs, which raises resting cytosolic calcium and increases PASMC proliferation via, among others, the Akt and NFAT signaling pathways (Song et al. 2018). Upregulation of the matricellular protein osteopontin by Sphingosine-1-phosphate (S1P) via calcineurin/NFAT signaling is observed in rat cell cultures (Yan et al. 2019). S1P induces a vasoconstrictive response, and via osteopontin, directly promotes PASMC proliferation.

Regulation of PAH by noncoding RNAs has also been shown in vitro, in particular miR-17-92 has been shown to regulate the PASMC contractile phenotype and increase proliferation in cells isolated from PAH patients (Chen et al. 2018).

#### Summary

PASMCs respond to increases in pulmonary arterial pressure by increasing vasoconstriction via the NFAT signaling pathway, increases in stiffness through proliferation mediated by miRNAs and dysregulated BMP and TGFβ1 signaling, and hypoxic conditions by depositing ECM proteins which remodels the pulmonary vasculature.

### Pulmonary arterial adventitial fibroblasts

PAAF cells are important for vascular ECM homeostasis and remodeling (Thenappan et al. 2018; Stenmark et al. 2006). There is evidence that PAAFs are regulated by matrix stiffness (Sun and Chan 2018; Dieffenbach et al. 2018; Dieffenbach et al. 2017), stretch (Strauss and Rabinovitch 2000), and hypoxia (Stenmark et al. 2006). In the presence of injury, PAAFs are activated and differentiate into myofibroblast subtypes that remodel vascular wall properties by altering the expression, degradation or cross-linking of ECM proteins including collagen, fibronectin and elastin (Stenmark et al. 2006). Given that the ECM also serves as a substrate for cell adhesion and sends physical and chemical cues that determine cell phenotype, it has recently been suggested that matrix remodeling and stiffening may themselves signal tissue remodeling and drive the disease process (Bertero et al. 2018).

#### In-vivo experiments

Balloon overstretch has been a useful way to study in vivo activation of PAAFs in injury. Juvenile swine had a high number of proliferating cells in the adventitia, and increased expression of PDGF, showing that adventitial myofibroblasts aid in lesion formation by synthesizing growth factors and alpha-smooth muscle actin (Scott et al. 1996). Another balloon overstretch experiment conducted by Mallawaarachchi et al. demonstrated that PAAFs are activated to myofibroblasts by stretch in PAH, migrate towards the lumen to form the neointima, and synthesize ECM after vascular injury mediated by the TGF-β1 pathway (Mallawaarachchi et al. 2005).

Hypoxia is also a critical regulator of matrix gene expression by activating ROS signaling that stimulates increased alpha smooth muscle actin (α-SMA) production, a marker of activated fibroblasts (Barman SA et al. 2014). Work by Chai et al. (2018) showed that hypoxia induces PAAF proliferation, migration, and vascular remodeling via the PI3K/Akt pathway, inducing medial and adventitial thickening and excessive fibronectin and collagen deposition in pulmonary artery walls of hypoxic rats in vivo.

In vivo studies have shown an overexpression of transient receptor potential vanilloid 4 (TRPV4), a calcium-permeable channel that is activated by mechanical stimulation, in chronic hypoxia and MCT rats (Cussac et al. 2020). In their study, Cussac et al. showed how upregulation of the TRPV4 channel leads to PAAF activation and adverse adventitial remodeling in PAH, increasing collagen I and fibronectin expression. Targeting TRPV4 could potentially ameliorate disease progression in PAH by reducing PAAF activation.

#### In-vitro experiments

In vitro experiments have shown the effect of altered substrate stiffness on PAAFs. Bertero et al. studied the YAP/TAZ and miR-130/301 vascular matrix feedback loop using cultured primary human PAAFs. They conducted quantitative RT-PCR of PAAFs on 1-kPa and 12-kPa stiffness matrices and found that stiffer matrices created a positive feedback loop, suggesting that matrix remodeling and stiffening may themselves signal tissue remodeling and drive the disease process (Bertero et al. 2018). The group also showed that YAP/TAZ signaling was activated by ECM stiffening from as low as 0.4 kPa to 1 kPa (Bertero et al. 2015). As part of the Hippo pathway, YAP/TAZ and the role of miRNA 130/301 have also been suggested to promote PAAF proliferation, collagen deposition and cross-linking (Dieffenbach et al. 2018). As the matrix stiffens due to fibrosis, angiotensin II is released by PAAFs activating pathways, such as the MAPK signaling cascade, that stimulate further ECM deposition (Boyd et al. 2011).

Recently, TRPV4 has been shown to mediate PAAF proliferation and migration in vitro using BrdU and wound scratch assays in PAAFs isolated from rats (Cussac et al. 2020). Cussac et al. showed how upregulation of TRPV4 activates PAAFs to transition to myofibroblasts. This work suggests a role for TRPV4 in excessive adventitial remodeling in PH.

Our group has shown how PAAFs respond to stretch and changes in extracellular matrix stiffness associated with PAH remodeling (Wang et al. 2021). PAAFs were differentially regulated by stretch and stiffness in expressing collagen I (*Col1a1*), collagen III (*Col3a1*), fibronectin (*Fn1*), α-SMA (*Acta2*), Lysyl oxidase-like 1 (*Loxl1*), and elastin (*Eln*) mRNA, but there were no interaction effects between stretch and stiffness for these genes. Increasing substrate stiffness resulted in an increase in collagen III and myofibroblast marker protein α-SMA, confirming the important role of PAAFs in PA stiffening and vascular remodeling. In addition, *Fn1* expression was significantly upregulated when PAAFs were stretched for 4 h but returned to baseline if cells were stretched for 24 h. However, *Col1a1* expression was only upregulated after PAAFs were stretched for 24 h. This faster response to stretch of *Fn1* than *Col1* suggests that more detailed timecourses of PA cell responses to stretch may be needed.

We used a sensitivity analysis of the computational model to predict which receptors would have the largest impact on downstream phenotypic outputs when inhibited. Based on this, we tested the effects of using losartan, an angiotensin II type I receptor inhibitor, on the relative expression of *Fn1*. Losartan inhibited the upregulation of *Fn1* by an increase in matrix stiffness, and revealed an angiotensin receptor-independent activation of *Fn1* expression by stretch in PAAFs grown on stiffer substrates.

#### In-silico experiments

We developed a computational model of PAAFs mechanosignaling that included TGFβ, MAPK, PDGF, tumor necrosis factor α (TNFα), hypoxia, fibroblast growth factor (FGF), angiotensin II, and Hippo signaling pathways, which are upregulated by PAH, and phenotypic outputs to investigate mechanical regulation of fibrosis in PAH (Wang et al. 2020). The model predicted 80% of the results from 20 independent papers not used for the original formulation. Sensitivity analysis showed PAAFs to be most sensitive to TGF-β1, MAPK and hypoxia signaling. In vitro, PAAF cells were cultured on hydrogel substrates having stiffnesses representing normal, mild and severe PAH vessels, and were subjected to biaxial cell stretch (Wang et al. 2021). Based on increasing the stimulus of the input “stiffness”, we ran experiments and verified the model-predicted upregulation of five profibrotic genes including *Col1a1*, *Col3a1*, and *Eln* mRNA in response to biaxial stretch, while six profibrotic genes including *Fn1* and *Acta2* were upregulated by increases in matrix stiffness in PAAFs. This computational framework allowed us to incorporate experimental findings, to predict how PAAFs would respond to inhibition of the angiotensin II and TGFβ receptors, and design new experiments. By using the model, we were able to successfully test our hypothesis of the differential effects of stretch and substrate stiffness in which stretch activates integrin-β3, Macrophage Stimulating 1 or 2 (MST1/2) kinases, angiotensin II, and the TRP pathways and stiffness activates integrin β3, MST1/2, angiotensin II, TGF-β1, and syndecan-4 signaling. Even though the model identified candidate pathways based on the available literature, this model was not able to replicate the observed inhibition of *Fn1* expression by losartan or the transient response of *Fn1* at 4 h seen in the Wang et al. (2021 study). In addition, while this model can accurately predict many independent experiments, it only predicts qualitative increases and decreases of expression. With more data, the predictive and quantitative power of this model should increase greatly.

#### Summary

PAAFs respond to hypoxia, stretch and stiffness by activating to myofibroblasts that produce more α-SMA, proliferate, and overproduce ECM proteins causing adventitial remodeling via YAP/TAZ and MAPK signaling in vitro and in vivo.

## Cellular crosstalk

**Fig. 4:**
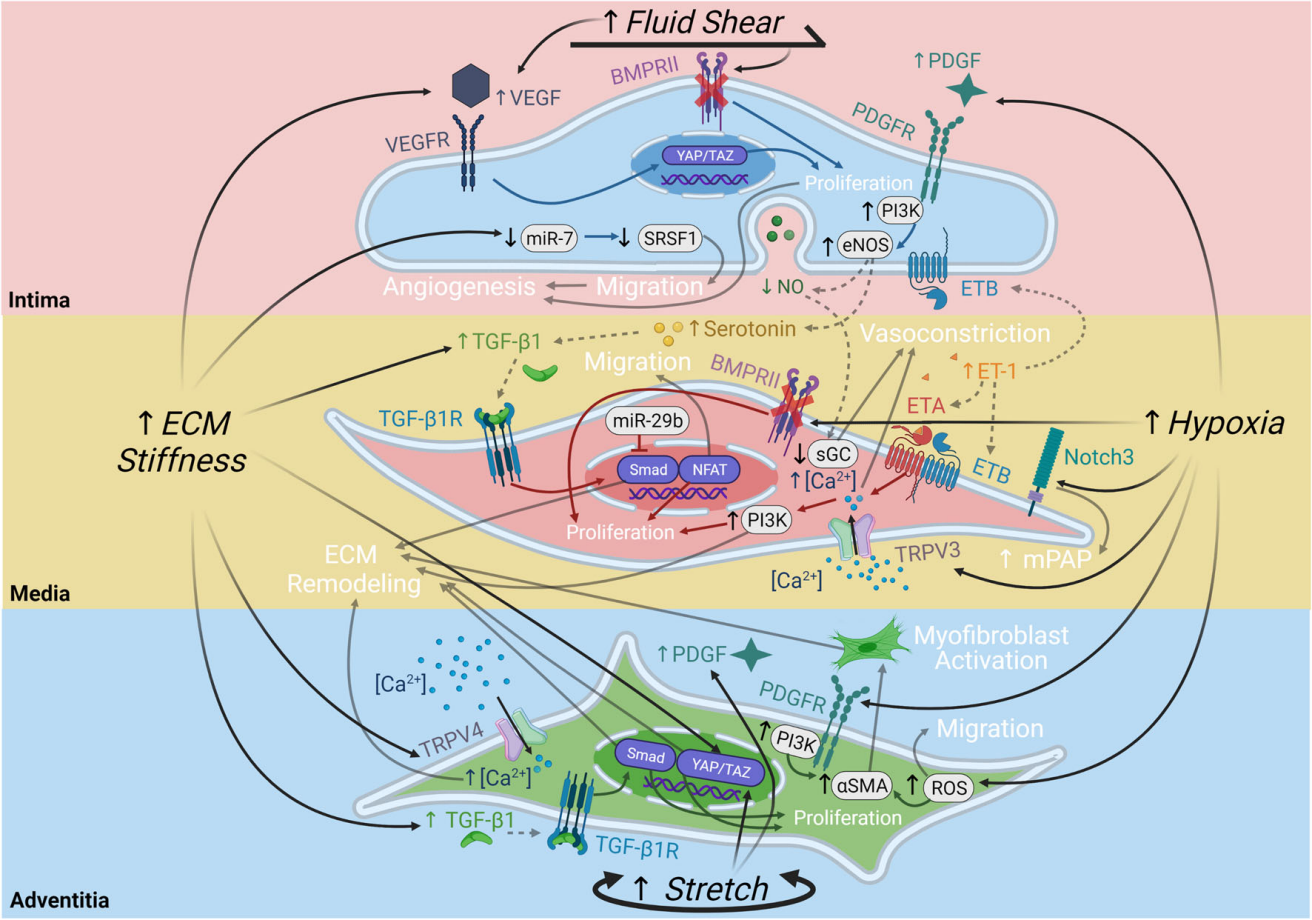
Intracellular and intercellular signaling pathways in pulmonary arterial cells regulated by fluid shear stress, stretch, hypoxia and extracellular matrix (ECM) stiffening. PAECs respond to fluid shear stress via vascular endothelial growth factor (VEGF) signaling and bone morphogenic protein (BMP) signaling, to ECM stiffness via VEGF signaling and miR-7, and to hypoxia via platelet-derived growth factor (PDGF) signaling. PASMCs respond to paracrine signals released by the PAEC and ECM stiffness via transforming growth factor beta 1 (TGF-β1), soluble guanylate cyclase (sGC), and endothelin (ET) signaling while responding to hypoxia via dysregulated BMP signaling and increased Notch signaling and transient receptor potential (TRP) channel calcium influx. PAAFs respond to ECM stiffness via TRP influx of calcium, TGF-β1 signaling, and activation of the Hippo pathway via YAP/TAZ transcription factors, to stretch via YAP/TAZ and PDGF signaling, and to hypoxia via reactive oxygen species (ROS) signaling. The four physical stimuli (black) lead to intercellular reactions (gray dashed arrows) and intracellular reactions (solid arrows matching cell color), resulting in phenotypic outputs (white). Illustration created with Biorender.com

In addition to the intracellular signaling via VEGF, BMP, PDGF, TGF-β1, Notch, endothelin and TRP channel signaling, mechanical crosstalk and paracrine signaling mediate interactions between PAECs, PASMCs, and PAAFs in response to physical stimuli caused by PAH, including altered fluid shear stress, stretch, ECM stiffness and hypoxia (Fig. 4).

Constriction of the pulmonary artery, regulated by PASMC contraction and relaxation, can be regulated by PAEC released paracrine factors. ECs release endothelium-derived constricting factors (EDCF), endothelin-1 (ET-1) and thromboxane A2 (TXA2), which lead to vasoconstriction via PASMCs (Makino et al. 2011). On the other hand, ECs can also release endothelium-derived relaxing factors (EDRF), NO and prostacyclin (PGI2), and the endothelium-derived hyperpolarizing factors (EDHF), which cause vasodilatation via PASMCs. This response between cell types are due to a connection of myoendothelial gap junctions, which transfer electric signals (Makino et al. 2011). Serotonin synthesized by PAECs is transferred through these gap junctions into PASMCs, where it activates TGF-β1 signaling and induces a more differentiated phenotype. As TGF-β1 is a crucial regulator of fibrosis, this is an important way PAECs and PASMCs respond to PAH (Gairhe et al. 2011, 2012).

A crucial way endothelium responds to stressors such as increased levels of TGF-β1, tumor necrosis factor-α (TNF-α), or interleukin-1β (IL-1β), is to undergo endothelial-to-mesenchymal transition (EndoMT). This cellular transdifferentiation results in some PAECs losing their endothelial markers, for example von Willebrand factor (vWF), and tight gap junctions between cells. The PAECs then express α-smooth muscle actin (α-SMA) and vimentin, fibronectin, and other markers of myofibroblasts. These transformed PAECs then go on to overproduce collagen I and other ECM proteins that remodel the matrix, inducing a profibrotic phenotype and increasing PA stiffness (Good et al. 2015). This then decreases vascular wall strain and further exacerbates ECM remodeling (Fig. 3).

### In-silico experiments

Recently, there has been published a computational model of the EndoMT transition integrating boolean equations, feedback mechanisms, and fixed patterns of activation which simulates cell behaviors and predicts effects of mutations. This allows them to explore conditions that cause EC activation, the transition, and reverse-transitions for future possible pharmacological control of the endothelial to mesenchymal transition (Weinstein et al. 2020).

There is currently no computational model of the crosstalk between PAECs, PASMCs, and PAAFs, or indeed any network models of PAECs or PASMCs individually, but a theoretical framework would allow us to synthesize experimental findings from different cell types and understand how their interactions contribute to pathological vascular remodeling.

### Summary

PAECs release paracrine factors that can affect the level of vasoconstriction and TGF-β1 signaling as mediated by PASMCs through gap junctions. Increased levels of TGF-β1 and TNF-α can lead to EndoMT wherein PAECs transform into activated PAAFs, or myofibroblasts, which increases deposition and accumulation of collagen and other ECM proteins (Fig. 4). This matrix remodeling can in turn increase ECM stiffness, which regulates PAEC migration and angiogenesis, matrix expression by PASMCs and PAAFs, and PAAF proliferation.

## Conclusions

There is now compelling evidence that mechanical cues caused by pulmonary arterial hypertension and subsequent disease remodeling regulate the responses of endothelial cells, smooth muscle cells, and adventitial fibroblasts in the pulmonary vasculature. As we continue to map the signaling pathways, paracrine interactions, and mechanical crosstalk in these cells, we can build predictive network models and use them to identify new therapeutic targets specifically to the mechanical milieu during different stages of the disease. It is likely that customized combination therapies could prove more effective than single targets in this context.
